# Physiological responses of *Agriophyllum squarrosum* and *Setaria viridis* to drought and re-watering

**DOI:** 10.1038/s41598-021-98246-8

**Published:** 2021-09-20

**Authors:** Juanli Chen, Xueyong Zhao, Yuqiang Li, Yongqing Luo, Yaqiu Zhang, Mei Liu, Yan Li

**Affiliations:** 1 Ecological Security and Protection Key Laboratory of Sichuan Province, Mianyang Normal University, Mianyang, 621000 China; 2 Naiman Desertification Research Station, Northwest Institute of Eco-Environment and Resources, Chinese Academy of Sciences, Lanzhou, 730000 China; 3 Hanzhong Agricultural Technology Extension Center, Hanzhong, 723000 China

**Keywords:** Evolution, Ecophysiology, Restoration ecology

## Abstract

Drought resistance of psammophyte determines survival and growth, but their responses to drought are not well understood. We conducted a pot experiment to study how physiological characteristics respond to drought and rehydration. We found that watering to 60–65% of field capacity (the control) provided more water than was required by *Agriophyllum squarrosum* and its leaves became yellow and slightly wilted. The total chlorophyll content and *F*m (maximum fluorescence after dark adaptation) in control were lower than in the drought treatment, and both decreased after rehydration. With increasing drought duration and intensity, the relative water content (RWC), chlorophyll content, *F*m, and the quantum efficiency of photosystem II (*F*v/*F*m) of *Setaria viridis* decreased, but malondialdehyde and membrane permeability increased. During the late drought, the activities of three antioxidant enzymes in *A. squarrosum* increased to prevent membrane lipid peroxidation; for *S. viridis*, only peroxidase and superoxide dismutase activities increased. After rehydration, RWC of both species increased, but *F*v/*F*m of *A. squarrosum* and *F*m of *S. viridis* did not recover under severe drought. Our research illustrated that *A. squarrosum* is better adapted to arid environment than *S. viridis,* but the high soil moisture content is not conducive to normal growth of *A. squarrosum*.

## Introduction

The world’s arid and semi-arid regions account for about one-third of the land area, versus nearly half of terrestrial land in China^1^. Water in these areas is unevenly distributed both temporally and spatially, and often does not satisfy plant needs^2,3^. Hence, plants are subjected to repeated cycles of drought and rehydration throughout their life cycle by this highly variable climate^4^. To understand plant survival under these conditions, it is necessary to comprehend the mechanisms that underlie plant physiological responses to drought and re-watering. With the global climate changing and the environment deteriorating, water shortages have become increasingly serious, causing a more intense situation on drought stress^5^. Drought causes water loss from plant cells, which leads to major changes in plant morphology, physiology, and biochemistry, and limits the growth range and living space of many species^6,7^. Many plants have evolved multiple mechanisms and strategies to adapt to water deficits through long-term natural selection and coevolution^8,9^.

Water availability limits vegetation in arid ecosystems by affecting photosynthesis, net productivity, and plant survive, both in natural vegetation communities and after ecological restoration^3,10^. Thus, dryland ecosystems respond rapidly to precipitation changes^11^. Studies have shown that plants osmoregulation can be carried out by accumulating osmotic regulator to maintain cell water balance, and our research about *A. squarrosum* and *S. viridis* is consistent with the results^12^. At the same time, many studies have indicated that plants can produce high levels of reactive oxygen species (ROS)under adversity, which results in increased membrane permeability (due to peroxidation of membrane lipids), protein inactivation, and even plant death^1,13^. The accumulation of ROS in plants also activates a protective system of antioxidant enzymes, with increased activities of antioxidant enzymes such as catalase, superoxide dismutase (SOD) and peroxidase, which scavenge excess ROS to maintain a balance between an active oxygen metabolism and reducing the damage to cell membranes caused by ROS^14,15^. Therefore, plant antioxidant enzyme defense function plays an important physiological role in plant stress resistance.

The effects of drought are intricate due to the different combinations of environmental characteristics and plant characteristics, including species differences^1,16^. Mafakheri et al.^17^ reported that drought dramatically decreased the contents of the chlorophyll a, chlorophyll b, and total chlorophyll content of three chickpea (*Cicer arietinum*) cultivars. It was also reported that drought stress reduced the chlorophyll fluorescence of olive, and that the decreased chlorophyll fluorescence in *Brassica rapa* resulted directly from a failure to recover from drought after rehydration^18^. However, drought promoted an increase in the chlorophyll content of *Periploca sepium*^5^. The ratio of variable to maximum quantum yield of PSII (*F*v/*F*m) in barley and sugar beet decreased under drought stress^19,20^, but the values of *F*v/*F*m and *F*m in chickpea remained stable^21^. Water deficits increased malondialdehyde and antioxidant enzyme activities in *Populus cathayana*^22^, but decreased the relative water content (RWC) in *Prunus avium*^23^. Membrane permeability in *Fargesia denudata* was weak owing to the higher activities of SOD and catalase^24^. The seed yield in *Glycine max* was positively correlated with SOD activity^25^, and there was a significant positive correlation between total antioxidant capacity and catalase activity^26^.

Horqin Sandy Land used to be famous grassland, but it has become the largest sandy land in China due to climate change, overharvesting and overgrazing^27^. During vegetation restoration to control the desertification, the succession that occurs in vegetation communities can be divided into three stages^28^: (1) Pioneer psammophyte such as *A. squarrosum* invades a site and colonizes the mobile dunes, the small and flat seeds of this species and the rapid growth of the radicle after germination help the seedlings to rapidly become established in the sand. (2) When the dunes have become semi-fixed, a community dominated by *Artemisia halodendron* has formed, and the vegetation biomass and cover result primarily from its perennial nature. (3) Stabilization of the land surface and the improved soil quality allow succession to climax species dominated by *S. viridis* and *Leymus chinensis* on the fixed dunes. The establishment of *A. squarrosum* plays a role in windbreak and sand-fixation, reduces the mobility of the surface matrix, and creates a stable matrix condition for the invasion of other species. The colonization by *A. halodendron* results in the accumulation of organic matter and the improvement of surface soil fertility. The distribution of *S. viridis* expands and its population increases greatly^29^.

Research on the physiological mechanisms that underlie stress resistance in psammophyte has mainly focused on survival of burial by sand, resistance to abrasion by the wind-sand flow, and endurance of drought stress^3,30–32^. To support preservation of natural vegetation or support ecological restoration, researchers who study psammophyte drought resistance have primarily focused on the short-term regulation of natural conditions or artificial transplanting, but it has been difficult to accurately describe the responses of psammophyte to drought^3^. The dominant species *A. halodendron* has been studied^1^ previously. However, researches on the response of the pioneer and climax species to drought stress are rarely discussed. Therefore, a study of the pioneer species *A. squarrosum* and the climax species *S. viridis* to drought was explored, and our objective was to reveal their adaptions to an extreme desert environment and to support the restoration of desert vegetation in a degraded sandy land. According to the previous research on the response of other species to stress, we hypothesized: (1) adequate water is favorable to the growth of *A. squarrosum* and *S. viridis,* but severe drought restrains the growth. (2) adequate water irritates the antioxidant enzyme activity, thus holding lower malondialdehyde levels and guarding plants from damages, but severe drought beyond their adjustable extent results in a remarkable augment of malondialdehyde and severe injury.

## Materials and methods

### Study area

The study area is located in the Naiman Banner of Inner Mongolia, near the edges of the Horqin Sandy land (42° 58′ N, 120° 43′ E; 360 m elevation). The region has a continental semi-arid monsoon climate. The annual mean temperature is 6.5 °C and annual potential evaporation average 1935 mm. This region receives 351.7 mm of precipitation annually, with 70–80% of the total in the growing season of June to August. The landscape is dominated by a mosaic of various types of dunes and sandy meadows. The zonal soil is a sandy chestnut soil, but the soil in most areas has degraded into an aeolian sandy soil due to the combination of a warm and dry climate with unsustainable human activities. The field capacity of the sandy soil was 13.0%. The main vegetation types are dune and meadow vegetation and the main shrubs are *Caragana microphylla*, *Salix gordejevii* and *A. halodendron*. The main herbaceous plants are *A. squarrosum*, *S. viridis*, *Artemisia frigida*, *Salsola collina*, *Cleistogenes squarrosa* and *Artemisia sieversiana*^12^. The experimental materials were *A. squarrosum* and *S. viridis*, and these species comply with institutional, national and international guidelines. The plant materials used in the study were previously isolated and identified by Halin Zhao in our laboratory. The specimens of *A. squarrosum* (Accession No: LZD0004353) and *S. viridis* (Accession No: LZD0001836) have been deposited in Northwest institute of Eco-environment and Resources, Chinese Academy of Sciences (http://www.nieer.cas.cn/).

### Experimental design and measuring

In August 2018 mature seeds of *S. viridis* were collected from fixed dunes. In October of the same year, *A. squarrosum* seeds were gathered. We sowed the seeds in plastic pots (28 cm in diameter and 21 cm in height) filled with 8.5 kg of air-dried and sieved (0.6 mm) dune soil at the end of April 2019. The young seedlings were thinned after 20 days of germination. Five seedlings of *A. squarrosum* and ten seedlings of *S. viridis* were retained in each pot after thinning. The remaining seedlings were healthy and similar in size. These plants were cultured under the same conditions and were irrigated with well water until the experiment began. The experiment was conducted at the Naiman Desertification Research Station, Chinese Academy of Sciences under a rain shelter to let us control soil water levels, but otherwise the plants grew under natural conditions. The shelter decreased the light intensity by about 10% below ambient levels, but was established at a height of 4 m so that the ambient temperature inside and outside the shelter differed by less than 1 °C.

In mid-July 2019, one hundred and twenty pots of each species were randomly separated into three water treatments: plants were watered to 60–65% of field capacity (the control), 40–45% of field capacity (moderate drought, MD), 20–25% (severe drought, SD) of field capacity. The severity was defined based on the physiological responses of *A. halondendron* to soil water in the previous study^1^. From 17 to 20 July, we regulated soil water content within the specified range based on the weight of soil in each pot. Subsequently, we maintained the water content within the range for 11 days (from 21 to 31 July) according to the field capacity (weight basis). On 1, 7 and 13 August (every 6 days after the end of water control), 60 pots were chosen (20 replicates each treatment selected randomly) to test the impacts of the sustained drought stress on the physiological indices. The remained 60 pots were well re-watered on 7 and 13 August to determine the effects of re-watering after sustained drought, and excess water flowed out of the hole at the bottom of the pot. After a day of rehydration (on 8 and 14 August), leaves were selected randomly to measure physiological responses. The experiment was repeated for two years and the results showed similar trends.

### Analytical methods and statistical analysis

On 1, 7, and 13 days after the initiation of drought treatments, we measured the physiological parameters of the fully developed leaves from the top of the plants selected from the first 60 pots. At the same time, the leaves of the same part as above from the rest 60 pots were chosen on the first day after rehydration on 8 and 14 August. The chlorophyll fluorescence (described below) was detected on the sampling date, and then the leaves were sampled to assay the degree of peroxidation of membrane-lipids and the activity levels of key antioxidant enzymes.

Chlorophyll was extracted from the leaves using 95% ethanol, and its content was determined at absorbance values of 665, 649 and 470 nm^33^. The chlorophyll fluorescence of dark and light adaptive leaves was stimulated, and determined with a portable chlorophyll fluorometer. (Hansatech, England)^34^. The minimum fluorescence with all photosystem II (PSII) centers open (*F*o) was measured under the condition of dark adaption for 30 min, and the maximum dark-adapted fluorescence (*F*m) was measured by saturation pulse irradiation at 2800 μmol/(m^2^ s). Variable fluorescence (*F*v) equaled the difference between *F*m and *F*o. The maximum efficiency of PSII equaled *F*v/*F*m^35^.

RWC = [(Fresh Weight − Dry Weight)/(Turgid Weight − Dry Weight)] × 100%. RWC was used to reflect leaf water status^36^.

Random 1 g leaves were extracted with buffer solutions and centrifugation, and the supernatant was stored at 4 °C for assaying malondialdehyde content and the activities of catalase, SOD, and peroxidase^1^. Catalase activity was expressed as the quantity of enzyme needed to eliminate 10^-6^ mol H_2_O_2_ in 1 min^37^. SOD activity was expressed as the quantity of enzyme needed to prevent the reducing of nitroblue tetrazolium^38^. Peroxidase activity was defined as the increase of the oxidation of guaiacol absorption of 0.01 one min^3^. The malondialdehyde content was quantified using the method of Heath et al.^39^. We have determined the above colorimetric analysis with UV-1601 UV–Visible Spectrophotometer (Shimadzu Corporation, Japan).

All data analyses were performed with the SPSS software (version 20.0) (http://www.ibm.com/software/analytics/spss/) and the graphs were drawn using version 12.5 of SigmaPlot (https://systatsoftware.com/). One-way analysis of variance (ANOVA) was performed to compare differences among the treatments. We used Fisher’s-least-significant-difference (LSD) test to detect significant differences between pairs of drought levels and used Pearson's correlation coefficient (r) to identify relationships among physiological features.

## Results

### Changes in chlorophyll content and fluorescence

**Figure 1:**
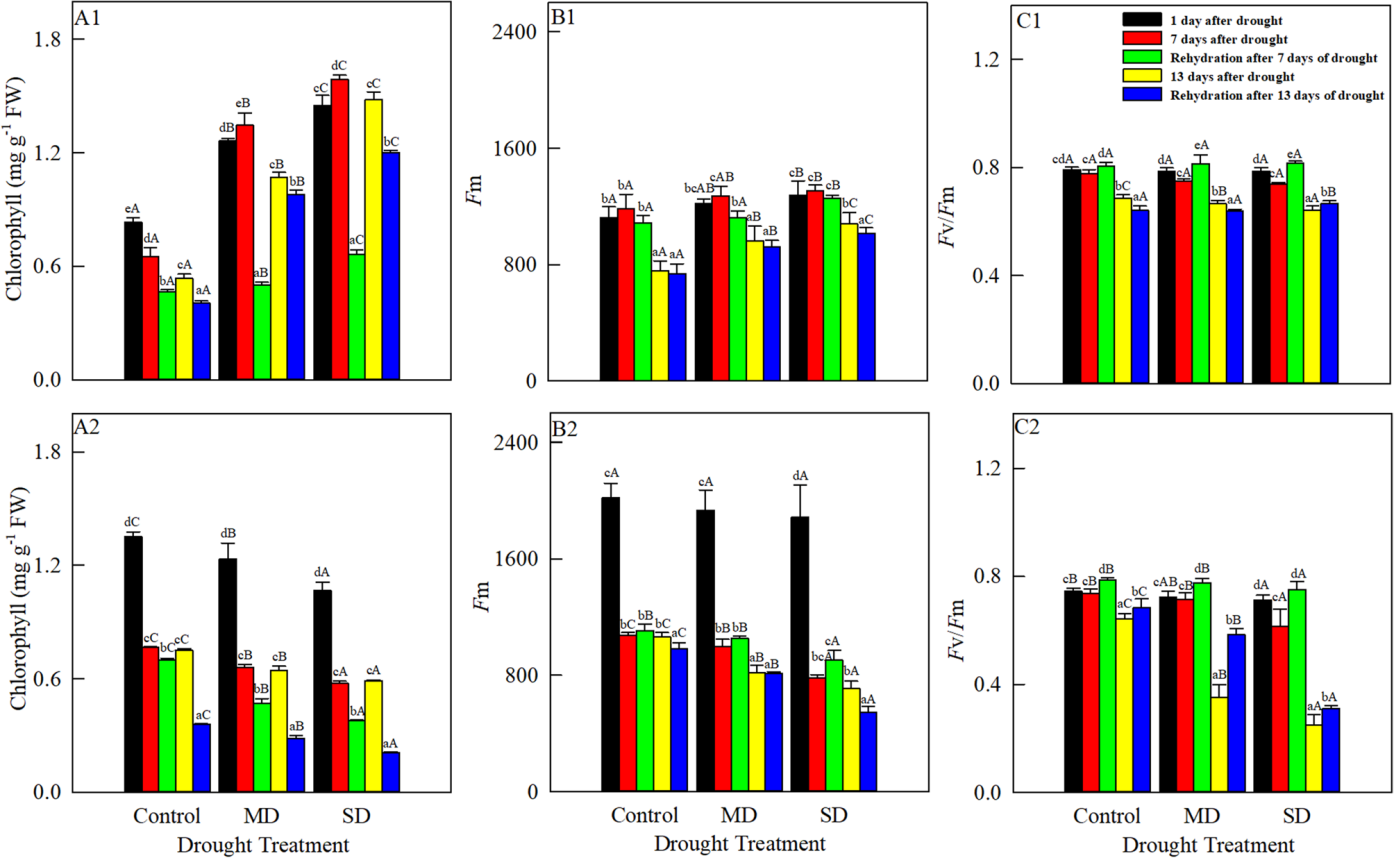
Changes in chlorophyll content, *F*m and *F*v/*F*m of *A. squarrosum* (**A1**, **B1**, **C1**) and *S. viridis* (**A2**, **B2**, **C2**) under drought stress (August 1, 7, 13) and rehydration (August 8, 14). Different lowercase letters indicate significant differences among days for the same treatment. Different capital letters indicate significant differences among drought treatments for the same day.

The total chlorophyll content of *A. squarrosum* in the control decreased gradually with increasing drought duration (Fig. 1A1), but the contents under moderate and severe drought first increased (from 1 to 7 August) and then decreased (from 7 to 13 August). During the same periods, the total chlorophyll content under moderate drought was significantly higher than that in the control, but significantly less than that under severe drought. The total chlorophyll content of *S. viridis* (Fig. 1A2) decreased with increasing drought duration and intensity, and the reduction from 1 to 7 August was greater than that from 7 to 13 August. After re-watering on 8 and 14 August, the total chlorophyll contents of both studied species were significantly lower than those before rehydration. In addition, the total chlorophyll content of *A. squarrosum* was in the order of severe drought > moderate drought > Control, and the order of *S. viridis* was severe drought < moderate drought < Control.

*F*m of *A. squarrosum* (Fig. 1B1) first increased (from 1 to 7 August) and then decreased (from 7 to 13 August) in all treatment. On 7 August, *F*m under severe drought reached the highest value (1309.1). In contrast, *F*m of *S. viridis* (Fig. 1B2) decreased as drought intensity and duration increased, and the reduction from 7 to 13 August was lower than that from 1 to 7 August. On 8 and 14 August, rehydration decreased the *F*m of *A. squarrosum*, and *F*m on 14 August was significantly lower than that on 8 August. On 14 August, *F*m was lowest in the control (735.4), which was 20.3% and 27.4% lower than those under moderate and severe drought, respectively. On 8 August, *F*m of *S. viridis* was higher than that before rehydration, but the opposite was true on 14 August.

With increasing drought duration and intensity*, F*v/*F*m of both species (Fig. 1C1, C2) slowly decreased (from 1 to 7 August) and then rapidly decreased (from 7 to 13 August). On 13 August, *F*v/*F*m was lowest under severe drought, and was significantly lower than in the control and under moderate drought. On 1 August, there was no significant difference in *F*v/*F*m of *A. squarrosum* among the treatments, but *F*v/*F*m of *S. viridis* was significantly higher in the control and under moderate drought than under severe drought. In addition to *F*v/*F*m of *A. squarrosum* in the control and under moderate drought on 14 August, rehydration led to a remarkable increase in *F*v/*F*m of both species on 8 and 14 August. *F*v/*F*m of *A. squarrosum* was in the sequence of severe drought > moderate drought > Control, and the sequence of *S. viridis* was severe drought < moderate drought < Control.

### Changes in RWC, malondialdehyde content and membrane permeability

**Figure 2:**
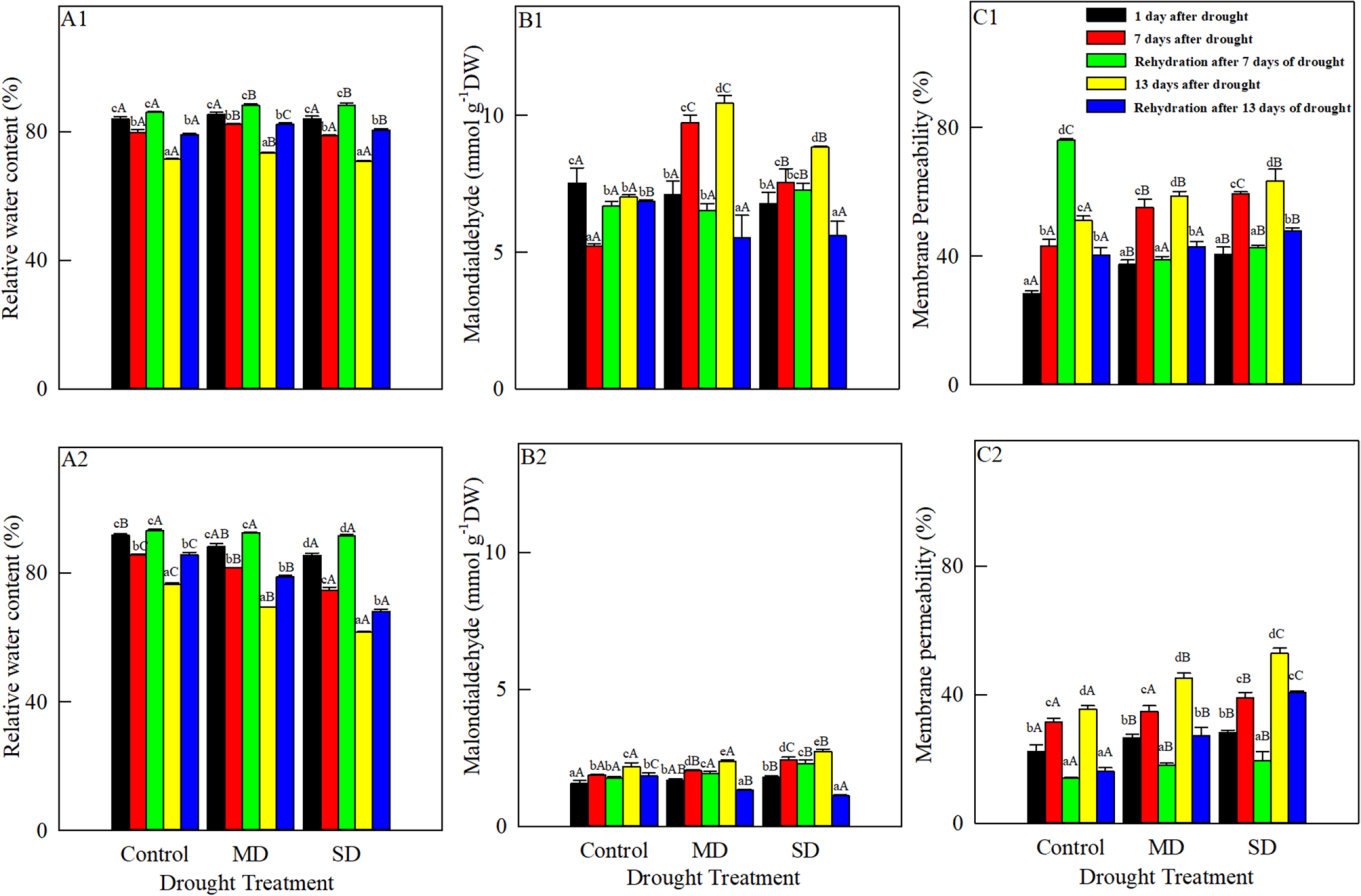
Changes in RWC, malondialdehyde and membrane permeability of *A. squarrosum* (**A1**, **B1**, **C1**) and *S. viridis* (**A2**, **B2**, **C2**) under drought stress (August 1, 7, 13) and rehydration (August 8, 14). Different lowercase letters indicate significant differences among days for the same treatment. Different capital letters indicate significant differences among drought treatments for the same day.

RWC of both species (Fig. 2A1, A2) decreased markedly from 1 to 13 August. RWC of *A. squarrosum* under moderate drought was significantly higher than those in the control and under severe drought. The RWC of *S. viridis* also decreased with increasing drought intensity. After re-watering on 8 and 14 August, RWC of both species were significantly higher than the corresponding values before rehydration and RWC on 8 August was observably higher than that on 14 August. RWC of *A. squarrosum* under severe drought was highest (88.2%) on 8 August, but RWC of *S. viridis* reached its highest value in the control (93.1%).

With increasing drought duration, the malondialdehyde content in the control of *A. squarrosum* (Fig. 2B1) first decreased (from 1 to 7 August) and then increased (from 7 to 13 August), but the content under moderate and severe drought increased continuously. The content decreased with increasing drought intensity on 1 August. However, the contents under moderate drought on 7 and 13 August were significantly higher than those in the control and under severe drought. The malondialdehyde content of *S. viridis* (Fig. 2B2) increased with increasing drought duration and stress, and reached the highest level under severe drought on 13 August (2.714 mmol g^−1^ DW). Although the malondialdehyde content in the control of *A. squarrosum* increased after rehydration on 8 August, the contents in both species on 8 and 14 August were lower than those before rehydration.

Membrane permeability of the two species (Fig. 2C1, C2) increased with increasing drought duration and stress. On 1 August, membrane permeability of *A. squarrosum* (28.2%) and *S. viridis* (22.2%) reached its lowest values in the control, and these values were significantly lower than those under moderate and severe drought. After re-watering on 14 August, rehydration reduced membrane permeability of *A. squarrosum* in the control, but permeability reached its highest level (63.3%) on 8 August, and this was significantly lower than the value before rehydration. On 8 and 14 August, membrane permeability of *A. squarrosum* under moderate and severe drought and of *S. viridis* under all treatments was lower than that before rehydration.

### Changes in the activity of antioxidase

**Figure 3:**
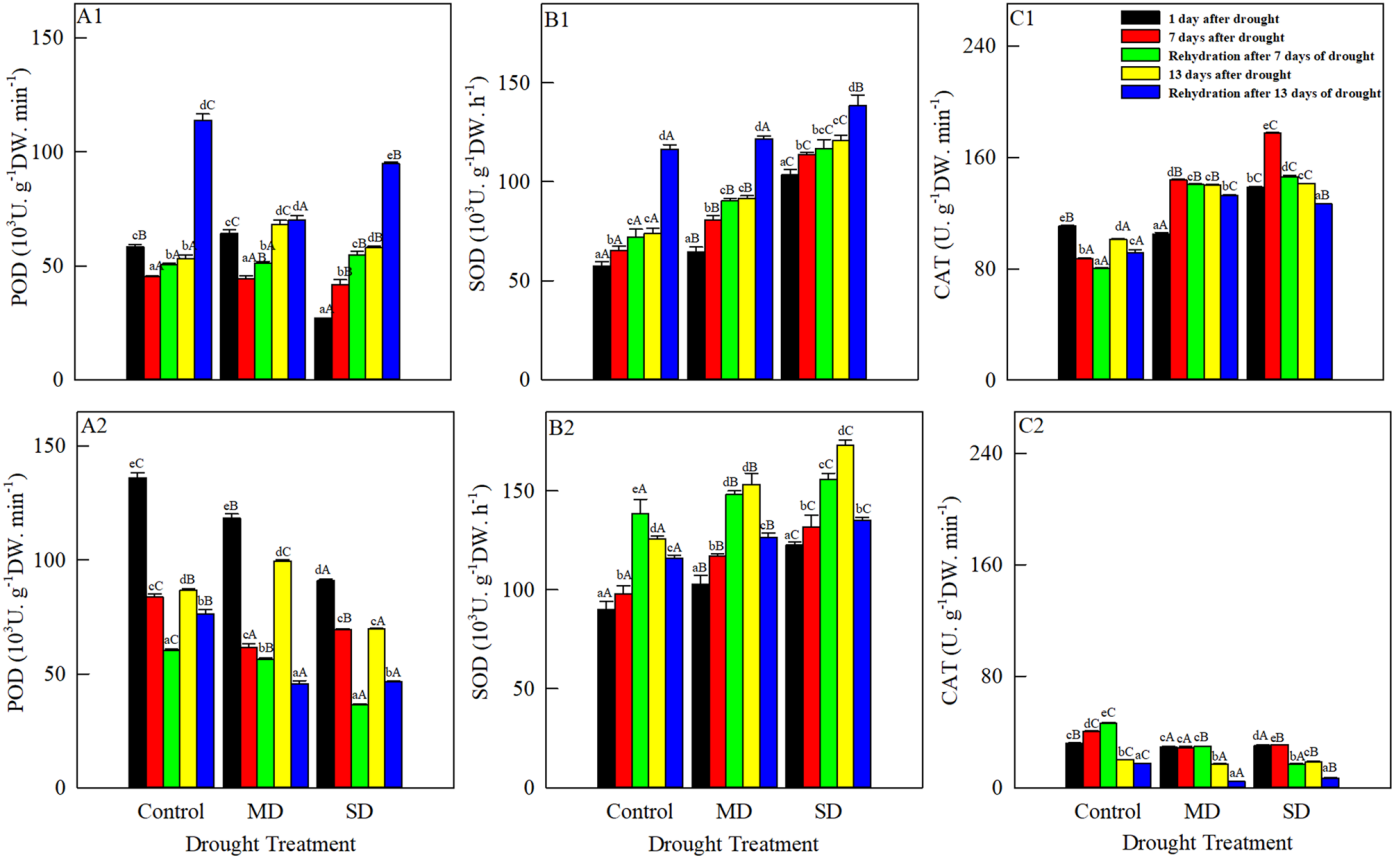
Changes in POD, SOD and CAT of *A. squarrosum* (**A1**, **B1**, **C1**) and *S. viridis* (**A2**, **B2**, **C2**) under drought stress (August 1, 7, 13) and rehydration (August 8, 14). Different lowercase letters indicate significant differences among days for the same treatment. Different capital letters indicate significant differences among drought treatments for the same day.

With increasing drought duration, peroxidase activities of both species (Fig. 3A1, A2) first decreased and then increased, except that the activity in *A. squarrosum* under severe drought increased continuously. Peroxidase activity of *S. viridis* was the largest (136.073 10^3^ U g^−1^ DW min^−1^) in the control on 1 August, 1.15 times and 1.50 times, respectively, the values under moderate and severe drought. After re-watering on 8 and 14 August, peroxidase activity of *A. squarrosum* was higher than that before rehydration, whereas *S. viridis* showed decreases after both re-hydrations.

SOD activities of both species (Fig. 3B1, B2) increased with increasing drought duration and severity. From 1 to 13 August, their activities were significantly higher under severe drought than in the control and under moderate drought. On 13 August, SOD activity of *S. viridis* was highest under severe drought, at 173.071 10^3^ U g^−1^ DW h^−1^, which was 1.13 and 1.38 times the value in the control and under moderate drought, respectively. SOD activity of *A. squarrosum* after rehydration was higher than that before rehydration, and its activity increased with increasing drought stress. On 8 August, rehydration increased SOD activity in *S. viridis*, but its activity decreased below that before rehydration on 14 August.

With increasing drought duration, catalase activity of the both species (Fig. 3C1, C2) first increased (from 1 to 7 August) and then decreased (from 7 to 13 August), but the catalase activity of *A. squarrosum* in the control showed the opposite pattern. On 1 August, catalase activity in the control of *A. squarrosum* was significantly higher than that under moderate drought, but was markedly and significantly lower than that under severe drought. On 7 August, its activity was highest under severe drought (177.416 U g^−1^ DW min^−1^). Catalase activity of *S. viridis* was low, at less than 50 U g^−1^ DW min^−1^ throughout the study period. On 8 and 14 August, rehydration caused an increase in catalase activity of the two plants, except that the activity in the control of *S. viridis* reached its highest level (46.425 U g^−1^ DW min^−1^) on 8 August.

### Correlation analysis

Many significant correlations were found among the physiological responses for both species (Table 1). For *A. squarrosum,* chlorophyll content was significantly positively correlated with *F*m and catalase, and catalase was significantly positively correlated with SOD, but peroxidase was significantly negatively correlated with *F*v/*F*m and *F*m. *F*v/*F*m was significantly positively correlated with *F*m and RWC, but negatively correlated with SOD. For *S. viridis*, *F*m and peroxidase were significantly positively correlated with chlorophyll content and *F*v/*F*m, and RWC was significantly positively correlated with *F*v/*F*m and *F*m. Catalase was significantly positively correlated with chlorophyll content and *F*v/*F*m, but Membrane permeability was negatively correlated with RWC and *F*v/*F*m. SOD was significantly positively correlated with malondialdehyde, but negatively correlated with peroxidase, total chlorophyll content, *F*v/*F*m, and *F*m.

**Table 1:** Values of Pearson’s correlation coefficient (*r*) for the relationships among the physiological characteristics for all drought durations, including values after rehydration, in *A. squarrosum and S. viridis*.

Species	Item	Total chlorophyll	*F*m	*F*v/*F*m	RWC	MDA	MP	POD	SOD
*A. squarrosum*	*F*m	0.520*							
	*F*v/*F*m	− 0.167	0.700**						
	RWC	− 0.226	0.497	0.749**					
	MDA	0.370	0.116	− 0.139	− 0.376				
	MP	0.155	− 0.006	− 0.197	− 0.375	0.360			
	POD	− 0.292	− 0.685**	− 0.618*	− 0.166	− 0.135	− 0.166		
	SOD	0.296	− 0.148	− 0.552*	− 0.154	− 0.102	0.121	0.410	
	CAT	0.657**	0.461	− 0.054	− 0.039	0.400	0.076	− 0.301	0.514*
*S. viridis*	*F*m	0.908**							
	*F*v/*F*m	0.366	0.549*						
	RWC	0.348	0.582*	0.936**					
	MDA	− 0.012	− 0.257	− 0.196	− 0.315				
	MP	− 0.097	− 0.403	− 0.835**	− 0.930**	0.424			
	POD	0.879**	0.761**	0.124	0.130	− 0.006	0.043		
	SOD	− 0.569*	− 0.633*	− 0.537*	− 0.456	0.542*	0.350	− 0.575*	
	CAT	0.576*	0.465	0.610*	0.550*	0.138	− 0.341	0.344	− 0.343

Abbreviations: *F*_m_, maximum chlorophyll fluorescence; *F*_v_, variable chlorophyll fluorescence; *F*_v_/*F*_m_, quantum efficiency of photosystem II; RWC, relative water content; MDA, malondialdehyde; MP, membrane permeability; POD, peroxidase; SOD, superoxide dismutase; CAT, catalase.

* and ** indicate a significant correlation at *P* < 0.05 and *P* < 0.01, respectively.

## Discussion

Chlorophyll plays an important part in the assimilation, transfer and conversion of light energy during photosynthesis. Its content is therefore closely related to the carbon fixation efficiency of photosynthesis and, because photosynthesis provides the energy source for metabolic responses, plays an important role in the drought resistance of plants. Chlorophyll fluorescence is often used to analyze photosynthesis efficiency under stress^1^. *F*m is the fluorescence output when the reaction center of PSII is completely closed, and therefore reflects the maximum electron transfer through PSII^40^. *F*v/*F*m represents the energy conversion efficiency of PSII reactions, and can be used to measure the degree of external stress^41^.

The chlorophyll content and *F*m of *A. squarrosum* first increased and then decreased under moderate and severe drought, indicating that *A. squarrosum* adjusted its energy capture during the early stage of drought, and because electron transfer was relatively stable, normal photosynthesis was maintained. As stress intensified during prolonged drought, chlorophyll degradation accelerated and electron transfer through PSII slowed, which was similar to the effect of drought stress on chlorophyll of *A. halodendron*^1^. On 1 August, when the drought treatments began, the leaves of *A. squarrosum* in the control became noticeably yellow and slightly wilted, and the chlorophyll content and *F*m were lower than those in the drought treatments. After re-watering, the chlorophyll content and *F*m of *A. squarrosum* decreased, but they increased with increasing drought intensity. There is a limit to plant demand for water, and both too much and too little water are not conducive to plant growth. As a pioneer species during vegetation succession in sandy land, *A. squarrosum* is a xerophyte^31^. The soil moisture content in the control was higher than its requirements, and its photosynthesis was obviously adversely affected by controlling the water content at a higher level than the plants required. There was a significant positive correlation between *F*v/*F*m and RWC of the two plants, which indicated that water deficit was the main reason for the decrease of *F*v/*F*m. The chlorophyll fluorescence of *A. squarrosum* could maintain higher photosynthetic performance under drought stress because of its stronger water holding capacity than that of *S. viridis*. For *A. squarrosum*, *F*v/*F*m decreased with increasing drought duration and intensity. This is because drought reduced the electron transfer capacity of PSII and photochemical activity, leading to excessive accumulation of excitation energy, and adversely affecting photosynthesis. *F*v/*F*m increased after re-watering on 8 August, when the reduction of stress slowed the inhibition of photosynthesis by drought, by decreasing the inhibition of photosynthesis.

For *S. viridis*, the chlorophyll content, *F*m and *F*v/*F*m decreased with increasing drought duration and intensity, indicating that drought stress hindered the biosynthesis of chlorophyll, and that chlorophyll decomposition increased, leading to decreased chlorophyll content. At the same time, drought resulted in the decrease of PSII photochemical transformation efficiency and photosynthetic activity, and the damage of PSII receptor, which contributed to the damage of photosynthesis and the decrease of electron transfer ability. *F*m and *F*v/*F*m of *S. viridis* increased after re-watering on 8 August, showing that rehydration relieved the drought stress. In addition, *F*v/*F*m increased and *F*m decreased after re-watering on 14 August, suggesting that the damage to PSII was mitigated by rehydration, but the electron transfer in the PSII reaction center continued to be slower than normal. The chlorophyll content of *S. viridis* did not return to normal after re-watering, indicating that the leaves of *S. viridis* were damaged by both prolonged and severe drought stress and that chlorophyll synthesis was significantly affected^1^.

The cell membrane is both a dynamic barrier between the cell interior and its surroundings, and a channel for the exchange of substance and information with its environment^42^. In particular, it controls water transport between the cell and its environment, leading to changes in RWC. RWC can be used to indicate the degree of dehydration of cells and assess the level of drought suffered by plants^43^. Under drought stress, the loss of water in plants is directly related to the stability of the cell membrane, and a stable cell membrane is the most basic requirement for maintaining sufficient water to support the cell’s physiological functions. ROS are produced in large quantities under stress, and this can trigger or exacerbate peroxidation of membrane lipids to produce malondialdehyde. Malondialdehyde can damage the membrane and functional molecules such as proteins and nucleic acids in cells, leading to damage or destruction of the membrane’s structure and functions. This, in turn, can increase the permeability of the membrane, leading to growth inhibition or even death. Therefore, changes in membrane permeability and the malondialdehyde content can reflect the degree of membrane lipid peroxidation and cell damage under stress^1,3,32^. It is consistent with our correlation analysis that RWC of the two species is negatively correlated with malondialdehyde and membrane permeability, and the correlation between RWC and membrane permeability in *S. viridis* is significant.

In *A. squarrosum*, membrane permeability in the control on 1 August was significantly less than those under moderate and severe drought, but the malondialdehyde content did not differ among the treatments. The change of membrane permeability may have resulted from degreasing of membrane lipids and destruction of the membrane structure after phospholipid dissociation^44^. From 1 to 13 August, malondialdehyde content of *A. squarrosum* in the control first decreased and then increased, while membrane permeability increased continuously, indicating that membrane lipid peroxidation was significantly alleviated in wet soil after short-term drought. In contrast, the severe water deficit during the late stage of drought increased peroxidation of membrane lipids and malondialdehyde accumulation, suggesting that the cell membranes in the control had been damaged during the drought process. The malondialdehyde content and membrane permeability of *A. squarrosum* increased in the control after rehydration on 8 August, but decreased after rehydration on 14 August. This suggests that rehydration during the early stages of drought can exacerbate the peroxidation of membrane lipids and damage the cell membrane, but that rehydration during the late stages of drought mitigated the stress and eased the damage. Many studies showed that membrane permeability and the malondialdehyde content increased synchronously under stress^1^, but this contradicts our results for *A. squarrosum* in the control. This may be because the high soil moisture content in the control was not conducive to normal growth of this xerophyte. That is, long-term natural selection in the species’ arid sandy environment would lead to continuous adaptation to its environment, allowing *A. squarrosum* to become widely distributed in the mobile dunes of the Horqin sandy land^45^. With increasing drought duration, the malondialdehyde content and membrane permeability of *A. squarrosum* increased under both moderate and severe drought, indicating that the accumulation of malondialdehyde after drought stress damaged cell membrane and increased its permeability. The RWC values of *A. squarrosum* in the control were similar, but the membrane permeability fluctuated greatly. This can be due to more than adequate amount of irrigation.

*Setaria viridis* is a late-successional species, and showed different responses to drought. With increasing drought duration and intensity, RWC of *S. viridis* decreased, while MDA and membrane permeability increased simultaneously. The results indicated that the early occurrence of water stress and membrane peroxidation in *S. viridis* under stress was one of the main physiological reasons for its inferior drought tolerance to *A. squarrosum*. Moreover, the damage degree of plants under drought stress should take into account not only the change of membrane permeability, but also the degree of membrane peroxidation and the ability of plant cell membrane to tolerate membrane lipid peroxidation. The chlorophyll content, *F*m and *F*v/*F*m of *S. viridis* decreased with increasing drought duration and severity, and *F*v/*F*m of *S. viridis* was significantly negatively correlated with membrane permeability, which increased with increasing drought stress. This indicated that membrane lipid peroxidation and the accumulation of ROS under drought stress damaged the membrane and inhibited photosynthesis. Re-hydration of *S. viridis* increased RWC on both dates and in all drought treatments. This was accompanied by decreased malondialdehyde content, particularly after the 14 August re-watering, and by decreased membrane permeability. Rehydration reduced membrane lipid peroxidation, but it did not return to the control level, showing that drought caused a certain degree of damage that may be permanent or that may take some time to be repaired^3^.

Stress can disrupt the balance of ROS metabolism in aerobic plants. When the concentrations of ROS are too high, peroxidation of membrane lipids and the equilibrium for exchanges of cell materials is also disrupted, resulting in a series of physiological and metabolic disorders. To counteract these disorders, plants have evolved protective enzymes during long-term evolution. The enzymes can eliminate O^2-^, H_2_O_2_, OH^-^ and O^-^ and reduce the damage they cause to the plant^46^. The changes in antioxidant enzyme activities of both species differed under drought stress. SOD played an active role during initial protection against membrane lipid peroxidation and its activity in *A. squarrosum* increased gradually during the drought. Under natural drought condition, SOD activities of the two species increased gradually, indicating that SOD activity was easily induced by drought stress. At the end of natural drought, the three enzymes of *A. squarrosum* maintained high level, and the combination of enzymes could resist drought stress, while only POD and SOD in *S. viridis* were enhanced to alleviate membrane lipid peroxidation. This transformation of the coordination of enzyme activity may be an important physiological mechanism of drought tolerance of *A. squarrosum* was stronger than that of *S. viridis* under severe drought. On 7 August, the peroxidase and catalase activities decreased in the control. Because ROS are a metabolism by-product of photorespiration, photosynthesis was inhibited by short-term drought, and the decreased accumulation of ROS caused by protective antioxidant enzymes reduced membrane lipid peroxidation by decreasing levels of malondialdehyde^47^. On 7 and 13 August, the activities of protective enzymes in *A. squarrosum* under moderate and severe drought were greater than that in the control. Drought stress led to the accumulation of ROS, and increased membrane lipid peroxidation, as reflected by the malondialdehyde content. At the same time, the accumulated ROS also stimulated the antioxidant enzyme protection system to continuously increase the activities of enzymes, so as to maintain balance of ROS^48^.

*Setaria viridis* showed different responses. From 1 to 13 August, its peroxidase activity first decreased and then increased, but catalase activity showed the opposite pattern, and SOD activity increased gradually, indicating the existences of coordination among these enzymes under drought stress^49^. When catalase activity weakened, SOD and peroxidase activities compensated for this weakness to scavenge more ROS and mitigate cell membrane damage. The catalase activity in *S. viridis* remained less than 50 U g^-1^ DW min^-1^ throughout the study. After rehydration, catalase activity in the control was significantly greater than those under moderate and severe drought. There was a close relationship between *F*v/*F*m and catalase activity in *S. viridis*. It is possible that the enzyme must be contributing through ROS scavenging. Some of the antioxidant enzymes of both species did not recover after rehydration, which may be related to the possibility that in xerophytes, rehydration did not immediately improve physiological metabolism. It is possible that their antioxidant enzyme systems were so damaged that they would take longer than our study period to return to normal levels, and our samples were collected1 day after rehydration.

## Conclusions

Hypothesis 1 and 2 were both partially demonstrated. Adequate water was not conducive to the growth and had negative effects on chlorophyll fluorescence and antioxidant enzymes. Severe drought had negative impact on these indices. Through the above analysis and discussion of the research, we concluded that *A. squarrosum* is better adapted to arid environment than *S. viridis*, which is why *A. squarrosum* can survive on mobile dunes, whereas *S. viridis* can only be colonizes when dunes become fixed dunes. With increasing drought intensity, its chlorophyll content and *F*m increased, whereas *S. viridis* showed the opposite trend. In addition to the control of *A. squarrosum,* malondialdehyde content and membrane permeability of both species enhanced synchronously with increasing drought duration, demonstrating adverse effects of drought stress. The activities of peroxidase, SOD and catalase in *A. squarrosum* collectively reduced membrane lipid peroxidation under drought stress, whereas *S. viridis* mainly alleviated membrane damage through peroxidase and SOD. Drought stress caused different degrees of damage to the two plants, and both failed to fully recover after rehydration during the study period, although full recovery might have occurred over a longer time. In future research, it will be necessary to clarify the stress resistance mechanisms of *A. squarrosum* and *S. viridis* by examining physiological responses such as stomatal transpiration and cellular processes such as adjustments in cell osmotic potential, turgor pressure, and compatible solutes.
